# Extrapolating Parametric Survival Models in Health Technology Assessment: A Simulation Study

**DOI:** 10.1177/0272989X20973201

**Published:** 2020-12-07

**Authors:** Daniel Gallacher, Peter Kimani, Nigel Stallard

**Affiliations:** Warwick Medical School, University of Warwick, Coventry, Warwickshire, UK; Warwick Medical School, University of Warwick, Coventry, Warwickshire, UK; Warwick Medical School, University of Warwick, Coventry, Warwickshire, UK

**Keywords:** cancer, extrapolation, health technology assessment, Monte Carlo simulation, survival analysis

## Abstract

Extrapolations of parametric survival models fitted to censored data are routinely used in the assessment of health technologies to estimate mean survival, particularly in diseases that potentially reduce the life expectancy of patients. Akaike’s information criterion (AIC) and Bayesian information criterion (BIC) are commonly used in health technology assessment alongside an assessment of plausibility to determine which statistical model best fits the data and should be used for prediction of long-term treatment effects. We compare fit and estimates of restricted mean survival time (RMST) from 8 parametric models and contrast models preferred in terms of AIC, BIC, and log-likelihood, without considering model plausibility. We assess the methods’ suitability for selecting a parametric model through simulation of data replicating the follow-up of intervention arms for various time-to-event outcomes from 4 clinical trials. Follow-up was replicated through the consideration of recruitment duration and minimum and maximum follow-up times. Ten thousand simulations of each scenario were performed. We demonstrate that the different methods can result in disagreement over the best model and that it is inappropriate to base model selection solely on goodness-of-fit statistics without consideration of hazard behavior and plausibility of extrapolations. We show that typical trial follow-up can be unsuitable for extrapolation, resulting in unreliable estimation of multiple parameter models, and infer that selecting survival models based only on goodness-of-fit statistics is unsuitable due to the high level of uncertainty in a cost-effectiveness analysis. This article demonstrates the potential problems of overreliance on goodness-of-fit statistics when selecting a model for extrapolation. When follow-up is more mature, BIC appears superior to the other selection methods, selecting models with the most accurate and least biased estimates of RMST.

## Introduction

### Background

In England and Wales, when a medical device or pharmacological developer wishes to get a technology approved for a new indication, they submit a report containing clinical and cost-effectiveness evidence concluding that their technology will offer cost-effective benefit to the National Health Service (NHS). Usually a submission will include an economic model, which captures the benefit of the intervention across a patient’s lifetime through the estimation of the mean survival time. Depending on the indication, this model may be required to extrapolate beyond the observed data to predict the future performance of the intervention and its comparators, as the data will often be heavily censored due to limited trial follow-up. The optimal extrapolation from data for time-to-event outcomes, such as overall survival, is routinely a point of contention within the decision making process and is often influential on the conclusion of the appraisal.

The National Institute of Health and Care Excellence (NICE) is responsible for deciding which treatments should be reimbursed by the NHS. Many have their clinical and cost-effectiveness assessed through the single technology appraisal (STA) process, where evidence submitted by a pharmaceutical company is appraised by an independent evidence review group prior to a discussion and decision by a NICE technology appraisal committee. The committee will consider whether the treatment is both clinically and cost-effective, with the latter assessed through the incremental cost-effectiveness ratio (ICER), which divides the incremental benefit of the new therapy by the incremental cost. The ICER can vary considerably based on the time-to-event extrapolation. If the most plausible ICER falls below certain thresholds, the treatment would usually be considered cost-effective and be recommended for reimbursement.

A recent review^1^ reported that the time horizon of economic models included in submissions to the NICE for cancer interventions in 2017 was on average 31.4 y (range, 10–100 y) and 15 times the median follow-up period, demonstrating the importance of reliable and robust methods for extrapolation.

### Methods for Model Selection

The most common approach to extrapolation in NICE appraisals is to select a single model from a range of parametric models (e.g., exponential).^1,2^ Candidate models are usually compared on 2 metrics: on the goodness of fit to the observed data and on the plausibility of the extrapolations. This article considers the former, since the plausibility of extrapolations can only objectively be demonstrated when suitably mature data are available. Plausibility would usually be assessed through a comparison of predictions of the percentage of patients who remain event free following a certain length of follow-up. Ideally, these assessments of plausibility are made comparing to relevant studies with longer observed follow-up, but if this is unavailable, then it is necessary rely on the opinions of clinical experts. While expert opinion from clinicians and experienced analysts may offer valuable insight into the plausibility of certain models and their extrapolations compared to known patterns of disease, these opinions may not always agree, especially when considering novel therapies.

Goodness of fit is often assessed visually (e.g., comparing models to Kaplan-Meier curves or cumulative hazard plots). While assessing visual fit may sometimes rule out particular models, it can be difficult to distinguish between most models due to their similar appearance, justifying a need for a less subjective approach. Hence, there is increased reliance on statistical comparisons to assess the goodness of fit.

**Figure 1:**
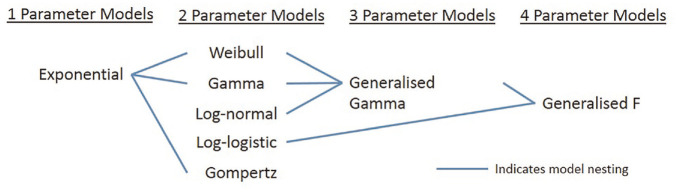
Nesting relationships among models fitted in this simulation study.

A well-established measure of model fit is the -2·log-likelihood statistic, where a lower score suggests a better fit. The statistic can compare 2 nested models in a formal hypothesis test, the likelihood ratio test. In this article, we compare both nested and nonnested models and so can only perform a simple comparison of the log-likelihood statistics. Figure 1 visualizes the nesting relationships for the models that we consider in this study. When comparing models with a large number of potential covariates, relying solely on the log-likelihood can result in the selection of an overfitted model. Although survival extrapolation models often exclude covariates, they may have different numbers of parameters due to the different parameterizations of the survival models. Models with more parameters may be at greater risk of overfitting.

Akaike’s information criterion (AIC) expands on the log-likelihood. AIC includes a penalty based on the number of parameters in the model, with the aim of selecting a parsimonious model, rather than the best-fitting model. When presenting his information criterion, Akaike demonstrated that relying on the maximized log-likelihood alone could bias the model selection but that this bias was approximately equal to the number of estimable parameters under certain conditions.^3^ The AIC is therefore defined as

(1) AIC=−2log(L)+2K,

where *L* is the likelihood and *K* the number of parameters in the model, meaning that the model with the lowest AIC is the closest of the candidate models to the true model.

The Bayesian information criterion (BIC), derived by Schwarz,^4^ uses a different weighting for the number of parameters, multiplying the penalty of the AIC by the natural logarithm of *n* to give

(2) BIC=−2log(L)+2Klog(n),

where *n* is either more commonly the number of observations, as in this article, or the number of events as recommended by Volinsky and Raftery.^5^ While the log-likelihood, AIC, and BIC can often agree on the best model, moving from log-likelihood to AIC to BIC will inevitably lead to an increased tendency to select a model with fewer parameters due to the increasing penalty.

Survival extrapolations influence the cost-effectiveness assessment through their estimation of either mean survival or restricted mean survival time (RMST). These outcomes are equivalent to life years used by NICE, which are scaled by quality-of-life utility values to estimate treatment effect in quality-adjusted life years. RMST is the area under the survival model up to the time horizon of the economic model from the relevant STA. If a survival model predicts that all patients will have experienced an event on or before the upper bound of time applied in the RMST, then the RMST will equal the mean survival time.

### Objectives

NICE technical support document (TSD) 14 contains recommendations issued by the NICE decision support unit for extrapolations of individual patient survival data.^6^ In its recommendations, TSD14 favors AIC and BIC over the use of the log-likelihood statistic, alongside consideration of the plausibility of extrapolations. Consequently, the review by Gallacher et al.^1^ found that 100% (*n* = 28) of cancer STAs in 2017 used at least 1 of AIC and BIC when selecting a model for survival extrapolation. However, it is unclear whether using AIC or BIC actually provides a benefit over log-likelihood in terms of accuracy of mean survival estimation, motivating this work.

The aims of this article are as follows:

(a) to demonstrate the performance of fitting parametric curves to trial data when estimating RMST,
(b) to identify the dangers of selecting the incorrect model for extrapolation when estimating RMST, and
(c) to identify whether there is any distinction between AIC, BIC, and log-likelihood when estimating RMST.

## Method

To compare the 3 model selection methods in terms of extrapolation accuracy, we conducted a simulation study, using the AIC, BIC, and log-likelihood to select a model to many simulated data sets, following aims, data generating mechanisms, estimands, methods and performance measures (ADEMP) reporting recommendations (Table 1).^14^ Data were generated from exponential, Weibull, and generalized gamma distributions, chosen as these parameterizations routinely feature in health technology appraisals and contain different numbers of parameters capturing varying degrees of flexibility of the hazard function.^1^ The parameters of these distributions and patterns of follow-up were based on 4 completed trials. The aim was not to generate scenarios that reproduced data identical to each of the trials or their hazard curves but to obtain 12 scenarios where the parameters came from trials and could be considered representative of evidence included in technology appraisals.

**Table 1:** Summary of Study following ADEMP Guidelines^14^

ADEMP Category	Response
Aims	To demonstrate the performance of fitting parametric curves to time-to-event data when estimating restricted mean survival time (RMST). To identify the dangers of selecting the incorrect model for extrapolation when estimating RMST. To identify whether there is any distinction between Akaike’s information criterion (AIC), Bayesian information criterion (BIC), and log-likelihood when estimating RMST.
Data-generating mechanism	Data were repeatedly sampled from exponential, Weibull, and generalized gamma distributions using parameters estimated from fitting models to re-created data from 4 phase III trials using a frequentist framework.
Methods	Data were censored using trial-specific parameters to replicate the maturity of data when the technologies were considered for reimbursement. Eight parametric curves were fitted to the data, and their RMST estimates and goodness-of-fit statistics were captured. The models preferred by each of AIC, BIC, and log-likelihood were compared.
Estimand	RMST
Performance measures	Bias, empirical SE, mean-squared error, Monte-Carlo standard error

ADEMP, aims, data generating mechanisms, estimands, methods, performance measures.

**Figure 2:**
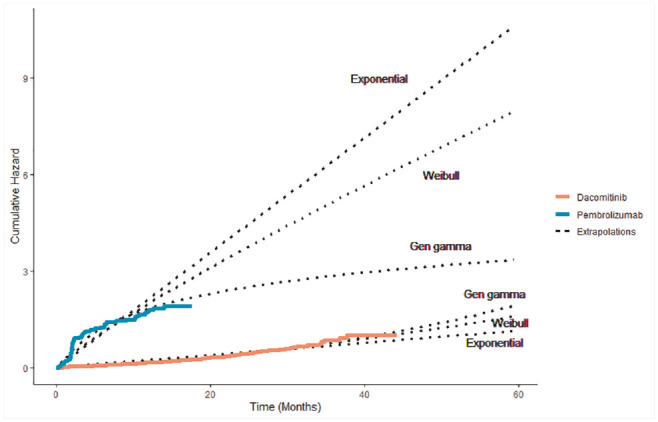
Observed and predicted cumulative hazard for pembrolizumab and dacomitinib trials.

**Figure 3:**
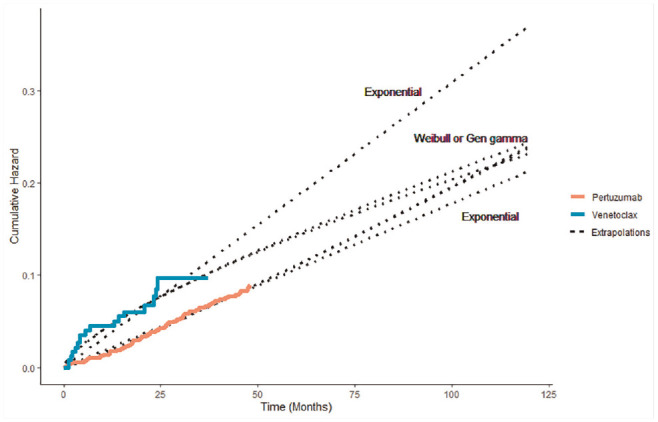
Observed and predicted cumulative hazard for pertuzumab and venetoclax trials.

We selected 4 trials that were pivotal in NICE STAs, covering a range of interesting scenarios, each with a differing combination of sample size, follow-up time, and number of events observed,^7–10^ focusing on their experimental arms. A summary of the trials is in Table 2, including the time horizon of each appraisal’s economic model used as the upper bound for estimating RMST. Additional detail of the parameters used to generate each scenario can be found in Supplemental Table S5. They are typical of phase III trials used in NICE technology appraisals, although characteristics will vary due to differences in diseases, prognosis, and outcome. The ARCHER 1050 trial had 103 overall survival (OS) events observed from 227 patients with epidermal growth factor receptor (EGFR)–mutated non–small cell lung cancer receiving dacomitinib in 46 months of follow-up, demonstrating an increasing hazard rate over time. In KEYNOTE 045, 437 progression-free survival (PFS) events were experienced across 542 second-line patients with advanced urothelial cancer in 18 months of follow-up, with the pembrolizumab PFS hazard rate clearly higher in the first 2 months of follow-up than for the remaining follow-up (Figure 2). APHINITY used the outcome of invasive disease-free survival, with 171 of the 2400 patients who had previously had human epidermal growth factor receptor 2 (HER2)–positive breast cancer experiencing events in the pertuzumab + trastuzumab arm in 48 months, with a constant rate of events observed. MURANO captured 15 OS events in 194 venetoclax + rituximab patients with relapsed or refractory chronic lymphocytic leukemia in 38 months of follow-up, demonstrating a constant or decreasing hazard rate (Figure 3).

**Table 2:** Summary of Clinical Trials Included in the Simulation

**Intervention**, *Trial*, and Indication	Outcome and No. Observed Events/No. Patients in Arm (%)	Median Survival	Maximum Follow-up	Description of Observed Hazard Behavior	How Company Modeled Outcome in Technology Appraisal	Time Horizon Used in Economic Model and to Calculate RMST	Censoring Parameters
**Dacomitinib** *ARCHER 1050* Treatment for untreated EGFR-positive non–small cell lung cancer	Overall survival 103/227 (45.4%)	34.1 mo	46 mo	Increasing hazard rate	Used a hazard ratio from a fractional polynomial model and applied to a generalized gamma extrapolation of a comparator	15 y (180 mo)	Recruitment period: 12 mo Recruitment λ: 0.2 Loss to follow-up λ: 0.002 Maximum follow-up: 18 mo
**Pembrolizumab** *KEYNOTE*-*045* Treatment for previously treated advanced or metastatic urothelial cancer	Progression-free survival 437/542 (79.2%)^a^	2.1 mo	18 mo	A sudden decrease in hazard rate at 2 mo	Used an exponential curve to data beyond 21 weeks	35 y (420 mo)	Recruitment period: 21 mo Recruitment λ: 0.1 Loss to follow-up λ: 0.002 Maximum follow-up: 48 mo
**Pertuzumab + trastuzumab** *APHINITY* Adjuvant treatment of early HER2-positive breast cancer	Invasive disease-free survival 171/2400 (7.1%)	Not reached	48 mo	Constant hazard rate	Used a log-logistic extrapolation	52 y (624 mo)	Recruitment period: 23 mo Recruitment λ: 0.1 Loss to follow-up λ: 0.002 Maximum follow-up: 46 mo
**Venetoclax + rituximab** *MURANO* Treatment for relapsed or refractory chronic lymphocytic leukemia	Overall survival 15/194 (7.7%)	Not reached	38 mo	Constant or decreasing hazard rate	Used a Weibull curve fitted jointly to both arms of the trial for both PFS and OS data simultaneously	20 y (240 mo)	Recruitment period: 18 mo Recruitment λ: 0.1 Loss to follow-up λ: 0.002 Maximum follow-up: 38 mo

EGFR, epidermal growth factor receptor; HER2, human epidermal growth factor receptor 2; OS, overall survival; PFS, progression-free survival; RMST, restricted mean survival time.

a Numbers are for whole trial, not reported by arm.

The 4 trials could be divided into 2 categories: trials where median survival was reached according to the Kaplan-Meier plot (dacomitinib/ARCHER 1050 and pembrolizumab/KEYNOTE 045) and those where the median was not observed (pertuzumab/APHINITY and venetoclax/MURANO).

The Kaplan-Meier curve for the novel intervention from each trial was digitized according to the method described by Guyot et al.^11^ to re-create individual patient-level data (IPD). Three different parametric models were fitted to each set of IPD to capture a variety of hazard profiles while maintaining a manageable number of simulation scenarios. Exponential, Weibull, and generalized gamma distributions were chosen due to their varying number of parameters and hazard flexibility. All models were fitted using Broyden-Fletcher-Goldfarb-Shanno optimization. The parameters from each of these 12 fitted models (i.e., 12 scenarios) were first used to calculate the “true” RMST for each scenario and second to generate 10,000 sets of time-to-event data via the rexp(), rweibull(), and rgengamma() commands included within the stats and flexsurv packages in R.^12^ Patients were simulated as being more likely to be recruited later in the recruitment duration, imitating the expanding recruitment of multicenter trials as more centers join a trial over time.

From each simulated set of data, 2 data sets were then created: the first with complete data where every individual had an observed event time and the second with event times censored for some individuals, mirroring the follow-up pattern observed in the actual trial. Censoring from incomplete follow-up was modeled by simulating a recruitment time for each patient from an exponentially increasing probability density function based on the recruitment period reported for each trial and a fixed trial end time given by the maximum follow-up time, with all patients without an event observed before this time considered censored. Additional censoring from loss to follow-up or withdrawal was modeled by generating censoring times for each patient from an exponential distribution with a low hazard rate.

### Analysis of Simulated Data

To each simulated set of data, we attempted to fit 8 parametric models: exponential, Weibull, log-normal, log-logistic, gamma, generalized gamma, Gompertz, and generalized F. The first 7 models feature routinely in technology appraisals,^1^ and the generalized F can be considered a generalization of the generalized gamma.^13^ The estimated value of RMST was recorded for each parametric model. Spline models were not included due to the additional specification required in selecting the optimal spline model. The best-fitting model in each simulation according to each of AIC, BIC, and log-likelihood was selected and the corresponding RMST recorded. Standard errors and confidence intervals around the RMST estimates are not easily obtained from fitted models and do not capture the uncertainty around the suitability of the survival curve. Reliance on these estimates of uncertainty that originate from the observed data may underestimate the structural uncertainty. For example, a confidence interval from a Weibull model will ignore the possibility the data could be better represented by a log-logistic model, which may only become clear once data from extended follow-up are available. The code used to simulate, fit models, and store estimates can be accessed at https://github.com/daniel-g-92/goodness-of-fit-paper1.

### Performance Measures

Bias of RMST estimates from the models was evaluated through calculation of the absolute and percentage difference of both the mean and median estimated RMST from the RMST value of the source distribution. Mean squared error (MSE), empirical standard error (EmpSE), and Monte-Carlo standard error (MCSE) of RMST estimates for all models were also measured, as defined by Morris et al.^14^ Coverage probability could not be estimated without confidence intervals of the individual RMST estimates. Instead, we provide an alternative measure, reporting the percentage of RMST estimates that fall within 10% of the target RMST value.

## Results

### Model Fitting

Only results from models that had successfully fitted without error or convergence warning were included in the analysis (Suppl. Table S1). Successful model-fitting rates were generally high, with only the trial scenarios of the generalized F and generalized gamma models having a success rate below 80%. The generalized F distribution often failed fitting to the pertuzumab data, fitting successfully in as few as 17% of runs. Due to the occasionally skewed distribution of the RMST estimates, the mean and median RMST estimates from the simulation runs are both presented. The Monte-Carlo standard errors were consistently minor compared to the RMST estimates, suggesting the number of simulations was suitable (Table 3 and Suppl. Table S4).

**Table 3:** Results for Trial Follow-up Scenarios^a^

Drug/Trial/Outcome/Source Distribution	True RMST (±10%)		Results										
		Measure	Exponential	Weibull	Log-Normal	Log-Logistic	Gamma	Generalized Gamma	Gompertz	Generalized F	Model with Lowest AIC	Model with Lowest BIC	Model with Highest Log-Likelihood
Pembrolizumab/KEYNOTE 045/PFS/exponential	5.6 (5.0 − 6.2)	Mean [bias] (5%, 95%) Median MSE MCSE EmpSE % within 10%	**5.61 [+0.01, +0%]** **(5.01, 6.28)** **5.60 [−0.00, −0%]** **0.15** **0.00** **0.39** **85.6%**	5.62 [+0.02, +0%] (4.99, 6.33) 5.59 [−0.01, −0%] 0.17 0.00 0.41 83.6%	9.10 [+3.50, +62%] (7.22, 11.58) 8.89 [+3.29, +59%] 14.12 0.01 1.38 0.2%	10.19 [+4.59, +82%] (8.32, 12.47) 10.06 [+4.46, +80%] 22.76 0.01 1.30 0.0%	5.61 [+0.01, +0%] (5.00, 6.30) 5.59 [−0.01, −0%] 0.16 0.00 0.40 84.8%	5.66 [+0.06, +1%] (4.97, 6.54) 5.61 [+0.01, +0%] 0.24 0.00 0.48 78.4%	6.11 [+0.51, +9%] (4.96, 8.86) 5.63 [+0.03, +1%] 4.72 0.02 2.11 70.3%	6.18 [+0.58, +10%] (5.04, 8.56) 5.84 [+0.24, +4%] 1.81 0.01 1.21 60.0%	5.97 [+0.37, +7%] (4.98, 8.75) 5.62 [+0.02, +0%] 3.05 0.02 1.71 76.9%	5.74 [+0.14, +3%] (5.00, 6.37) 5.60 [−0.00, −0%] 1.90 0.01 1.37 83.7%	6.17 [+0.57, +10%] (5.03, 8.58) 5.83 [+0.23, +4%] 1.91 0.01 1.26 60.8%
Pembrolizumab/KEYNOTE 045/PFS/Weibull	5.8 (5.2 − 6.4)	Mean [bias] (5%, 95%) Median MSE MCSE EmpSE % within 10%	5.51 [−0.33, −6%] (4.87, 6.18) 5.49 [−0.35, −6%] 0.27 0.00 0.40 71.0%	**5.87 [+0.02, +0%]** **(5.09, 6.75)** **5.84 [−0.01, −0%]** **0.26** **0.01** **0.51** **76.1%**	11.25 [+5.41, +93%] (8.41, 14.94) 11.00 [+5.16, +88%] 33.33 0.02 2.01 0.0%	12.48 [+6.64, +114%] (9.83, 15.65) 12.33 [+6.49, +111%] 47.22 0.02 1.78 0.0%	5.73 [−0.11, −2%] (5.02, 6.50) 5.70 [−0.14, −2%] 0.22 0.00 0.46 77.9%	5.93 [+0.09, +1%] (5.03, 7.10) 5.85 [+0.00, +0%] 0.43 0.01 0.65 68.1%	14.64 [+8.80, +151%] (5.38, 35.62) 11.14 [+5.30, +91%] 180.25 0.10 10.14 19.1%	6.86 [+1.02, +17%] (5.15, 10.90) 6.25 [+0.40, +7%] 4.93 0.02 1.97 48.0%	8.12 [+2.28, +39%] (5.08, 22.58) 5.92 [+0.08, +1%] 46.29 0.06 6.41 59.9%	7.79 [+1.95, +33%] (5.00, 23.65) 5.77 [−0.08, −1%] 47.98 0.07 6.65 65.6%	6.99 [+1.15, +20%] (5.14, 11.27) 6.23 [+0.39, +7%] 8.89 0.03 2.75 49.0%
Pembrolizumab/KEYNOTE 045/PFS/generalized gamma	12.0 (10.8 − 13.2)	Mean [bias] (5%, 95%) Median MSE MCSE EmpSE % within 10%	5.16 [−6.84, −57%] (4.52, 5.84) 5.14 [−6.86, −57%] 46.95 0.00 0.40 0.0%	5.22 [−6.78, −56%] (4.53, 6.00) 5.19 [−6.80, −57%] 46.14 0.00 0.45 0.0%	6.16 [−5.84, −49%] (5.07, 7.44) 6.10 [−5.90, −49%] 34.61 0.01 0.73 0.0%	7.31 [−4.69, −39%] (5.84, 9.04) 7.23 [−4.76, −40%] 22.93 0.01 0.98 0.1%	5.12 [−6.88, −57%] (4.46, 5.84) 5.10 [−6.90, −58%] 47.52 0.00 0.42 0.0%	**12.58 [+0.58, +5%]** **(7.67, 19.23)** **12.05 [+0.05, +0%]** **13.38** **0.04** **3.61** **26.3%**	30.22 [+18.23, +152%] (9.46, 54.73) 29.04 [+17.05, +142%] 524.08 0.14 13.85 3.8%	13.50 [+1.51, +13%] (7.96, 20.85) 12.98 [+0.98, +8%] 18.47 0.04 4.03 23.8%	12.83 [+0.84, +7%] (7.69, 19.93) 12.26 [+0.26, +2%] 15.30 0.04 3.82 25.4%	12.45 [+0.45, +4%] (6.27, 19.32) 12.08 [+0.08, +1%] 15.17 0.04 3.87 25.8%	13.48 [+1.48, +12%] (7.93, 20.82) 12.97 [+0.97, +8%] 18.41 0.04 4.03 23.9%
Pertuzumab/APHINITY/IDFS/exponential	376.8 (339.1 − 414.5)	Mean [bias] (5%, 95%) Median MSE MCSE EmpSE % within 10%	**377.08 [+0.24, +0%]** **(353.71, 401.26)** **377.13 [+0.29, +0%]** **207.85** **0.14** **14.42** **99.1%**	373.33 [−3.51, −1%] (311.12, 429.13) 375.14 [−1.70, −0%] 1299.50 0.36 35.88 70.7%	474.94 [+98.10, +26%] (444.34, 502.29) 475.73 [+98.89, +26%] 9936.52 0.18 17.69 0.1%	409.19 [+32.35, +9%] (363.46, 450.91) 410.28 [+33.43, +9%] 1754.38 0.27 26.61 55.7%	374.54 [−2.31, −1%] (321.78, 424.14) 375.31 [−1.54, −0%] 967.52 0.31 31.02 77.4%	377.52 [+0.68, +0%] (249.62, 489.12) 385.11 [+8.27, +2%] 7044.03 0.87 83.93 28.8%	357.48 [−19.36, −5%] (149.15, 540.86) 373.90 [−2.95, −1%] 18922.31 1.36 136.20 13.2%	359.51 [−17.34, −5%] (48.74, 517.28) 420.89 [+44.05, +12%] 24553.06 3.67 155.78 3.2%	367.97 [−8.87, −2%] (117.63, 487.51) 379.14 [+2.30, +1%] 10148.39 1.00 100.35 67.3%	361.49 [−15.35, −4%] (241.18, 401.84) 376.04 [−0.80, −0%] 5463.82 0.72 72.31 93.1%	362.26 [−14.58, −4%] (117.63, 512.31) 396.59 [+19.74, +5%] 15976.12 1.26 125.56 26.3%
Pertuzumab/APHINITY/IDFS/Weibull	338.7 (304.8 − 372.6)	Mean [bias] (5%, 95%) Median MSE MCSE EmpSE % within 10%	381.15 [+42.49, +13%] (356.83, 405.38) 381.00 [+42.35, +13%] 2021.77 0.15 14.70 28.1%	**336.85 [−1.81, −1%]** **(270.45, 400.14)** **338.09 [−0.57, −0%]** **1562.08** **0.39** **39.48** **60.3%**	459.73 [+121.07, +36%] (425.61, 491.04) 460.53 [+121.88, +36%] 15055.39 0.20 19.92 0.0%	382.73 [+44.08, +13%] (333.71, 429.59) 383.57 [+44.91, +13%] 2793.72 0.29 29.17 34.9%	345.42 [+6.77, +2%] (291.58, 399.12) 345.62 [+6.97, +2%] 1115.72 0.33 32.71 68.4%	340.62 [+1.97, +1%] (191.83, 476.34) 348.60 [+9.94, +3%] 9878.10 1.04 99.37 19.9%	234.05 [−104.60, −31%] (117.91, 481.43) 189.75 [−148.90, −44%] 24065.06 1.15 114.56 7.6%	336.43 [−2.22, −1%] (48.99, 504.25) 398.36 [+59.70, +18%] 23070.07 3.60 151.91 2.1%	326.83 [−11.83, −3%] (54.09, 461.40) 373.29 [+34.63, +10%] 15028.77 1.22 122.03 23.0%	353.99 [+15.33, +5%] (54.09, 410.03) 379.16 [+40.51, +12%] 8354.30 0.90 90.11 25.4%	311.79 [−26.87, −8%] (54.09, 481.47) 356.16 [+17.50, +5%] 18870.91 1.35 134.73 13.9%
Pertuzumab/APHINITY/IDFS/generalized gamma	333.5 (300.2 − 366.9)	Mean [bias] (5%, 95%) Median MSE MCSE EmpSE % within 10%	381.40 [+47.87, +14%] (357.92, 405.76) 381.06 [+47.53, +14%] 2504.79 0.15 14.59 15.9%	336.71 [+3.18, +1%] (269.31, 400.80) 337.99 [+4.46, +1%] 1595.29 0.40 39.82 58.9%	459.82 [+126.29, +38%] (425.58, 490.91) 460.78 [+127.25, +38%] 16347.79 0.20 19.99 0.0%	382.65 [+49.12, +15%] (332.78, 429.96) 383.63 [+50.09, +15%] 3278.31 0.29 29.42 28.5%	345.40 [+11.87, +4%] (290.55, 399.17) 345.59 [+12.05, +4%] 1227.17 0.33 32.96 65.2%	**337.86 [+4.32, +1%]** **(185.86, 477.76)** **343.81 [+10.28, +3%]** **10242.31** **1.05** **101.12** **19.7%**	233.09 [−100.44, −30%] (117.75, 486.77) 188.28 [−145.25, −44%] 23496.66 1.16 115.80 7.0%	335.31 [+1.78, +1%] (49.09, 501.25) 392.29 [+58.76, +18%] 22273.48 3.57 149.28 2.0%	325.00 [−8.54, −3%] (53.25, 462.89) 372.56 [+39.03, +12%] 15274.16 1.23 123.30 17.8%	352.75 [+19.22, +6%] (53.25, 409.24) 379.14 [+45.60, +14%] 8806.67 0.92 91.86 14.8%	309.11 [−24.42, −7%] (53.25, 483.50) 350.94 [+17.41, +5%] 19095.08 1.36 136.02 12.1%

AIC, Akaike information criterion; BIC, Bayes information criterion; EmpSE, empirical standard error; IDFS, invasive disease-free survival; MCSE, Monte-Carlo standard error; MSE, mean squared error; OS, overall survival; PFS, progression-free survival; RMST, restricted mean survival time.

a Shaded cells indicate model or model selection method with lowest MSE. Bold cells indicate model distribution matches source distribution.

### Model Extrapolation without Selection

There were similarities between the results within the groups based on whether median survival times had been observed (dacomitinib/ARCHER 1050 and pembrolizumab/KEYNOTE 045) or not (pertuzumab/APHINITY and venetoclax/MURANO), and so for brevity, we describe only results for pembrolizumab and pertuzumab trials here, with results for the other trials included in the supplementary information.

#### Pembrolizumab/KEYNOTE 045

For the exponential and Weibull scenarios of complete follow-up, most models fitted equivalently well. The exponential, Weibull, gamma, generalized gamma, Gompertz, and generalized F were all accurate, producing estimations of RMST within 10% of the true RMST in at least 80% of simulations and having the lowest EmpSE (Suppl. Table S4). Despite the maturity of the data, the log-normal and log-logistic models produced biased estimates of RMST for these scenarios, with the RMST estimates falling within 10% of the true value in 4.4% of simulations or fewer. The mean estimates of RMST from the log-normal and log-logistic models overestimated by as much as 70% (log-logistic fitted to Weibull pembrolizumab data; Suppl. Table S4), which corresponded to a difference of 4 months of additional overestimated benefit. MSE was low for all models apart from log-normal and log-logistic.

In the generalized gamma scenario of complete follow-up, the estimates of RMST showed considerable variation, with the generalized gamma and generalized F models being the best fitting, with the least bias, lowest MSE and EmpSE, and the most estimates of RMST within 10% of the true RMST. The log-logistic, log-normal, and Gompertz models were the worst fitting, with 3% to 7% of simulations producing estimates of RMST within 10% of the true value and their mean estimates of RMST underestimating by 28% to 30%. The gamma and exponential curves had extremely high MSEs and produced the most heavily biased estimates.

For the exponential scenario of trial follow-up, the exponential model was the most reliable predictor of RMST, with estimates falling within 10% of the true value in 86% of simulations but was closely followed by the Weibull (84%) and gamma (85%) models. The estimates from each of these 3 models were unbiased and associated with the lowest MSE and EmpSE. The log-normal and log-logistic were again biased with their mean estimates of RMST overestimating by 62% and 82%, respectively, and were associated with considerably higher MSE and EmpSE. This poor performance of the log-normal and log-logistic models was repeated in the Weibull scenario of trial follow-up, where the respective mean estimates of RMST were 93% and 114% over. However, the Gompertz model also produced biased estimates of RMST in the Weibull scenario, overestimating by 151%. Here the Weibull model was the least biased, but with the gamma model having slightly lower MSE and EmpSE.

All models produced estimates of RMST that varied highly for the generalized gamma scenario of trial follow-up. MSEs for all models were considerably higher than for the Weibull and exponential scenarios. The generalized gamma fit produced the least biased and most accurate estimates of RMST, with its estimates falling within 10% of the true value in 26% of simulations and mean estimate of RMST only 5% higher than the true value. The generalized F model was second best with slightly higher bias (+13%) and just 24% of estimates falling within the 10% range. For most other fitted models, 0% of RMST estimates fell within the 10% range. The mean RMST estimates for exponential, Weibull, log-normal, log-logistic, and gamma models all severely underestimated RMST, ranging from 39% to 57% below the true value. In contrast, the estimate from the Gompertz model overestimated by 152%.

#### Pertuzumab/APHINITY

For all 3 scenarios of complete follow-up, the majority of the models produced accurate estimates of RMST. The worst-fitting model was the log-normal, but even in the exponential scenario, where it was the least accurate, its estimates of RMST still fell within 10% of true value in 98% of simulations, and its mean RMST estimate was 8% less than the true RMST. In the pertuzumab scenarios of full follow-up, which had a much larger sample size than the pembrolizumab trial, the magnitude of the bias from any fitted model never exceeded 7%.

For the exponential scenario of trial follow-up, the exponential model was superior, with 0% bias, the lowest MSE and EmpSE, and with 99% of RMST estimates falling within 10% of the true value. The least accurate models were the Gompertz, generalized F, and log-normal, with 13%, 3%, and 0% of their respective estimates falling within 10% of the true RMST. Log-normal RMST estimates were the most biased, with the mean estimate 26% above the true value.

The models for the Weibull and generalized gamma pertuzumab scenarios were almost identical (Figure 3) and hence had similar results. The Gompertz, generalized F, and log-normal models performed poorly across these 2 scenarios, with less than 8% of the estimates falling within 10% of the true RMST and bias of up to 38%. The Weibull, generalized gamma, and generalized F models were unbiased in both scenarios. The gamma curve was associated with the lowest MSE and EmpSE and the highest percentage of estimates falling within the 10% range. Both MSE values and EmpSE were much higher for all models in the trial scenarios than the scenarios of complete follow-up.

### Model Selection

For all scenarios of complete follow-up, the models preferred by each of AIC, BIC and log-likelihood all produced near identical, unbiased estimates of RMST though BIC was slightly superior in the majority of cases (Table S4). For most scenarios of complete follow-up, the methods had similar estimates of MSE, MCSE and EmpSE. Only in the exponential scenario of pertuzumab were the methods distinguishable, with models preferred by BIC having the lowest EmpSE and MSE.

#### Pembrolizumab/KEYNOTE 045

**Figure 4:**
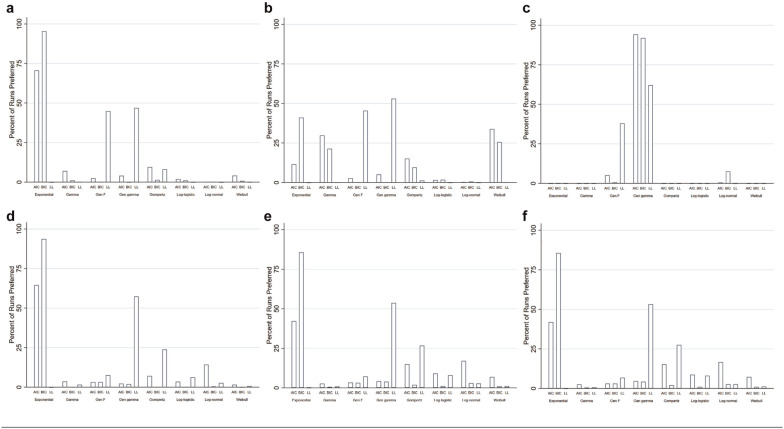
Distribution of curve selection. (a) Exponential pembrolizumab scenario. (b) Weibull pembrolizumab scenario. (c) Generalized gamma pembrolizumab scenario. (d) Exponential pertuzumab scenario. (e) Weibull pertuzumab scenario. (f) Generalized gamma pertuzumab scenario.

In the exponential scenario of trial follow-up, AIC selected the exponential model in 71% of simulations, compared to 96% for BIC and 0% for log-likelihood (Figure 4a). Models preferred by BIC were associated with the least bias (+3%), had the lowest MSE, and had the most estimates falling within the 10% range (84%). Models preferred by log-likelihood had the highest bias of the 3 selection methods (+10%) and the lowest percentage of estimates within the 10% range (61%) but the lowest EmpSE. Models preferred by BIC were only slightly outperformed by selection of the exponential model each time.

For the Weibull scenario of trial follow-up, the Weibull model was preferred by AIC in 34% of simulations. BIC and log-likelihood preferred Weibull in 26% and 0% of simulations, respectively (Figure 4b). Models with the highest log-likelihood were associated with the least bias (+20%), lowest MSE, and EmpSE but had the lowest percentage of estimates within 10% of the true value (49%). Models preferred by BIC had the highest percentage of estimates within the range (66%).

The methods of model selection were outperformed by repeated selection of either the Weibull or gamma model, which had less bias and more reliable RMST estimates, suggesting the selection methods could be improved.

In the generalized gamma scenario of trial follow-up, AIC favored the generalized gamma model in 94% of simulations, compared to 92% and 62% for BIC and log-likelihood, respectively (Figure 4c). Models preferred on the basis of BIC were associated with the least bias (+4%), lowest MSE, and EmpSE and had the most estimates within 10% of the true RMST (26%). The performance of the other 2 selection methods was similar. Selecting the generalized gamma curve every time would have only had a negligible improvement over relying on the methods of model selection, suggesting the selection methods did well.

#### Pertuzumab/APHINITY

From the simulations of the exponential scenario of trial follow-up, AIC selected the exponential model in 65% of the time, compared to 94% and 0% for BIC and log-likelihood, respectively (Figure 4d). Models with the lowest BIC were associated with the lowest EmpSE and MSE, as well as had the highest percentage of estimates in the 10% range, but models preferred by AIC had slightly less bias (+2%). Selecting the exponential model every time would have offered a small improvement over using BIC preferred models.

In the Weibull scenario, AIC, BIC, and log-likelihood preferred a Weibull model in 7%, 1%, and 1% of simulations, respectively (Figure 4e). Models with the lowest BIC had the lowest MSE and EmpSE, as well as the largest number of estimates in the 10% range (25%), but models preferred by AIC had slightly less bias (–3%).

Selecting the Weibull or gamma models would have considerably increased the percentage of estimates falling within the 10% range but had little impact on bias.

For the generalized gamma scenario, AIC and BIC favored the exponential (42% and 86%, respectively; Figure 4f) over the generalized gamma model (5% and 4%, respectively), whereas the log-likelihood favored the generalized gamma in 53% of simulations. Models preferred on the basis of AIC featured the least bias (–3%) and had the largest number of estimates within the 10% range (17.8%). Models preferred by BIC were associated with the lowest MSE and EmpSE. Selecting the generalized gamma model in every simulation would have had marginal improvements over using a method of model selection, but selecting the gamma model each time would have significantly increased the percentage of estimates falling within 10% of the true value (65%).

#### Agreement of AIC, BIC, and log-likelihood

We also explored whether greater reliability from AIC, BIC, and log-likelihood preferred models might be obtained if the approaches agreed on the same model. In most scenarios explored, there was almost no agreement on the preferred model between log-likelihood and BIC.

For the pembrolizumab exponential scenario, RMST estimates were no more accurate when AIC and BIC agreed on the best model. Of the 7471 simulations in which the 2 criteria agreed on the model, 6227 simulations fell within 10% of the true RMST value (83%), which was not an improvement on selecting the exponential model each time (86%) or on the BIC alone (84%) but did improve on the AIC alone (77%). Similar results were observed for the other source distributions and trials.

### Summary of Results

If a parametric model is selected that does not resemble the underlying survival distribution, it is likely to result in bias, even if data are complete.

Simple hazard behavior (exponential/constant) can be captured well from data simulating trial follow-up, but Weibull and generalized gamma often cannot.

In the scenarios considered, BIC was generally superior for selecting models that resulted in accurate and unbiased estimates of RMST when follow-up was suitably mature.

With immature data, all 3 methods can be biased in their model selection, leading to biased estimates of RMST.

In the scenarios of trial follow-up, the larger EmpSE associated with the models by each of the selection methods and the generalized gamma and generalized F models suggest that there remains considerable variation in the RMST estimates generated by these methods.

## Discussion

We have presented a range of scenarios demonstrating the dangers of extrapolating using parametric models and relying heavily on AIC, BIC, and log-likelihood for model selection. The incorrect choice of model can result in large bias, but even the model matching the underlying distribution can be associated with large variability in the RMST estimates, suggesting that extrapolating across population lifetimes may be a less than ideal tool for assessing the utility of health technologies. Model selection based solely on goodness-of-fit statistics has been shown to lead to bias due to the selection of the incorrect model. Incorporating some measure of plausibility may improve this by removing implausible models from consideration. BIC had a tendency to select the exponential model, which is the model with the fewest parameters, even when the source distribution was Weibull or generalized gamma, whereas the log-likelihood favored the generalized gamma and generalized F models, with the largest number of parameters, across all scenarios. When the follow-up was more mature, BIC appeared superior to the other methods, even when the source distributions contained multiple parameters. This could be influenced by the source distributions and parametric models considered but could be generalizable to scenarios beyond this simulation study. This result could also be driven by the fact that the set of candidate models included the true model. BIC has been shown to work better when this is the case, whereas AIC does not seek to identify the true model but the best predicting model.

It is clear that reliance solely on statistical measures of goodness of fit can result in severe bias and incorrect estimation of RMST even when 2 or more measures might agree on the optimal model, and it is reassuring that model plausibility is also usually considered in practice, although not always.^2^ Our scenarios of trial follow-up suggested that the models preferred by the selection methods were often associated with significantly higher MSEs than the best parametric model, suggesting there is room for improvement over AIC and BIC when selecting models for extrapolation. We have shown that the difference in the underlying assumptions of the shapes of the models is an important consideration, given that the log-normal, log-logistic, and Gompertz models often provided markedly difference estimates of RMST compared to the other parametric forms, particularly in the scenarios of trial follow-up. Of course, if we repeated our simulation study using these distributions to generate scenarios, then we would anticipate them to perform much better. We recommend that pharmaceutical companies provide very rigorous justification for their preferred model, perhaps prespecifying the model for extrapolation, including evidence of why the long-term hazard behavior can be considered plausible for their intervention. We have demonstrated that selection should not be based on goodness-of-fit statistics alone, and health technology assessors should be suspicious and perhaps automatically reject cases where no further consideration beyond information criteria is made. Even when follow-up is complete, selecting the incorrect parametric model may result in a biased estimate of mean survival.

We have shown that trial follow-up typical of that used in NICE technology appraisals can be insufficient for reliable extrapolation, even when the model selected from extrapolation matches that of the underlying sample distribution. It is concerning to see how the models and methods of model selection struggled with these relatively simple scenarios of time-to-event data, where all events came from a single distribution. As medical technologies advance and we move toward personalized medicine, it will be increasingly common to require greater flexibility than that which is offered by a single parametric model. We anticipate that the accuracy and reliability of extrapolations will only deteriorate as data come from more complex underlying hazard behaviors, especially when the behavior of the hazard rate is expected to differ beyond the observed period. Despite the fact that the scenarios considered here were relatively simple, all using single parametric distributions as a source, the fitted models often contained considerable uncertainty. It is likely that real-world data will not follow such simple forms, and it is possible that using the selection methods and parametric models may perform even worse in terms of either bias or uncertainty. Inevitably, there will often be key differences between high-risk patients whose events are observed and the low-risk patients remaining at risk after typical follow-up of a phase III trial, meaning the observed hazard rate behavior will not be representative of the hazard rate beyond the observed period. Even if the right model is selected, it is possible that the tail data will not contain enough information to accurately estimate the parameters. Alternatively, the hazard rate may deviate from the shape of all of the candidate models. While models incorporating cure proportions or time-varying hazard ratios sometimes feature in health technology appraisals, the data are likely to be insufficient to accurately estimate the parameters of these more complex models to provide reliable extrapolations. Reliance on goodness of fit to observed data will only be reliable when the unobserved hazard rate is linked to the observed behavior in a way that is captured by at least one of the candidate parametric models. By definition, each of AIC, BIC, and log-likelihood assesses model fit solely on the observed period, explaining why the performance of the methods improves as the length of follow-up increases. The methods of selection do not account for the uncertainty beyond the observed follow-up period. The suitability of parametric model extrapolation and goodness-of-fit statistics will surely vary across diseases and the patterns of treatment response.

Given the generally high levels of uncertainty when extrapolating (which will still exist when plausibility is taken into account), this raises the question of whether NICE should seek an alternative approach rather than rely on potentially inaccurate and unreliable extrapolations to decide whether to fund new interventions. If a NICE appraisal committee decides to focus on a particular subgroup of patients, rather than the licensed population that would usually be represented in the trial, the relevant survival data will contain even less information, inflating the uncertainty associated with RMST. Perhaps greater consideration of extrapolation should be made when designing clinical trials. There may be plenty of evidence of the long-term outcomes for a population receiving a comparator, in which case more patients could be recruited into the intervention arm of a trial, maximizing the opportunity to collect information necessary to obtain the most reliable extrapolation. If this is not feasible, perhaps recommendations should be made on a temporary basis, with a final decision on approval and pricing only made after a certain degree of follow-up has been observed, such as the current operation of the Cancer Drugs Fund in England. Alternative approaches that assess benefit on observed periods may encourage longer follow-up and reduce the uncertainty for decision makers but would probably slow down access to new therapies.

While the exponentially distributed trial data generally contained enough information for their underlying hazard to be captured by the fitted exponential models, the same is not true for the generalized gamma models. This questions whether it is appropriate to select a generalized gamma or generalized F model for extrapolation from typical follow-up of a phase III trial, given that their RMST estimate is not likely to be within 10% of the true RMST, as seen in 3 of the 4 modeled trials. This could be true even if it is known that the generalized gamma is representative of the population’s survival profile and may be driving the superiority of BIC. This may mean that simpler models should be preferred when data are particularly immature. The curves with more parameters also demonstrated overfitting to data simulated from a simpler distribution. The convergence issues experienced with the generalized F may explain its lower popularity in NICE appraisals. Heinze et al.^15^ have discussed the related concept of events per variable.

It is interesting how the estimates from the pertuzumab/APHINITY trial were associated with the most uncertainty, yet the company in this appraisal extrapolated using a parametric model as done in this study. Meanwhile, in the venetoclax appraisal, the company did not extrapolate their outcome, citing insufficient follow-up. It is unclear what truly motivates a company to select a method of extrapolation (e.g., whether to use parametric models or not), but it is possible that there is insufficient consideration of associated uncertainty, with greater emphasis placed on the resulting ICER.

Aside from the exponential distribution, all models have multiple parameters. It is possible that any bias or uncertainty is associated more strongly with some parameters than others or that the uncertainty associated with one parameter is more influential on the estimates of RMST. If this was true, it could alter the way that uncertainty around survival is accounted for in technology appraisals. Probabilistic sensitivity analyses (PSAs) are inconsistent with their inclusion of survival parameters and commonly exclude them.^1^ Identification and inclusion of key parameters could improve the uncertainty captured in PSAs. Ideally, a PSA would also include the uncertainty around the choice of model, but there is no well-established method for incorporating structural uncertainty into a PSA.

### Strengths

The strengths of this study include that it has captured the characteristics of 4 independent clinical trials that were highly influential in NICE technology appraisals. We have captured a range of different time-to-event outcomes, each with its unique combination of events, follow-up length, and hazard profile. The methods included are representative of current practice, and clear issues are identified. It is highly relevant to decision makers and disease modelers.

### Limitations

A simulation study of this kind is unable to verify the plausibility of every extrapolation or assess the visual fit of multiple models through a comparison to the Kaplan-Meier or cumulative hazard plot, and so we did not consider plausibility in our study. Both visual fit and extrapolation plausibility are commonly used alongside an information criterion when selecting a parametric model in practice, as recommended by 2 reviews,^1,2^ and may improve the application of the information criterion when used in combination. They could rule out clearly implausible extrapolations for a specific disease or indication, preventing these models from being selected by AIC and BIC. Similarly, the issues around convergence for certain models in particular scenarios may be introducing bias into the analysis. The results are also dependent on the models used to generate each scenario.

We have used RMST rather than overall mean survival time with the restriction applied corresponding to the time horizon used in each appraisal’s economic analysis. Following this truncation, the bias of RMST estimates for certain models may be underestimated. If we had allowed the time horizon to vary based on the predictions of each model, we would likely observe even more uncertainty in the estimates of RMST due to the tails of models such as the log-normal being allowed to carry on for much longer.

We used an arbitrary measure of 10% distance from the true RMST to measure accuracy of the RMST predictions. This allowed some degree of comparison across the different trials but may still be inappropriate due to the wide range in underlying RMSTs. For example, there may be much more uncertainty in the ICER in the venetoclax study, despite more of its RMST estimates falling within the 10% threshold across the various scenarios.

We have only considered RMST from single arms of trials. While potential bias should always be avoided, its effect may be reduced if the bias is roughly equivalent on both arms of a trial, hence having a minimal effect on the estimate of incremental differences in RMST. However, the broad uncertainty demonstrated in these simulations offers no guarantees that bias will be evenly distributed across trial arms. The level of uncertainty observed in the extrapolations also raises the question of whether extrapolation is an acceptable approach to estimating treatment benefit.

Only the characteristics of 4 trials were considered in this study. It is possible that the usefulness of AIC and BIC may vary based on the hazard profile, the selection of models considered, and the degree of follow-up.

We have simulated from only 3 parametric distributions. Real-world data may come from other distributions or may not be well represented by a parametric curve. Similarly, it is unclear whether alternatives to parametric modeling, such as combining trial data with external data^16^ or using Bayesian model averaging,^17^ may improve RMST predictions.

Relying solely on estimates of RMST alone, without assessment of plausibility at certain benchmarks, could mean that a seemingly unbiased estimate of RMST is the result of a combination of over- and underprediction. The consequences of this could be significant in economic modeling due to additional considerations such as discounting and age-related disutilities, meaning that it is important to capture when treatment benefit occurs, rather than just overall absolute benefit.

## Conclusion

This study demonstrates issues with parametric extrapolation of time-to-event data and the reliance on AIC and BIC to select a preferred model. We show that data from trials used in NICE technology appraisals are often inadequate to obtain an accurate and unbiased extrapolation. In scenarios of mature trial follow-up, BIC was generally superior to AIC and log-likelihood. Further work is needed to identify when data are mature enough to provide acceptable levels of certainty around survival extrapolations.

## Recommendations

Consider underlying hazard assumption, as assuming the incorrect model can result in large biases.

All model selection methods occasionally led to preferred models with extremely high estimates of mean survival. Care should be taken to also consider plausibility of estimates and to not solely rely on statistical goodness of fit when selecting a model.

BIC appears superior for model selection when data are sufficiently mature.

Restricted follow-up can contain too little information for a reliable model of a multiparameter model to be estimated and extrapolated.

## Supplemental Material

sj-docx-1-mdm-10.1177_0272989X20973201 – Supplemental material for Extrapolating Parametric Survival Models in Health Technology Assessment: A Simulation Study Click here for additional data file. Supplemental material, sj-docx-1-mdm-10.1177_0272989X20973201 for Extrapolating Parametric Survival Models in Health Technology Assessment: A Simulation Study by Daniel Gallacher, Peter Kimani and Nigel Stallard in Medical Decision Making

## References

[1] Gallacher D Auguste P Connock M. How do pharmaceutical companies model survival of cancer patients? A review of NICE single technology appraisals in 2017. Int J Technol Assess Health Care. 2019;35(2):160–7. 10.1017/S0266462319000175 31017564

[2] Bell Gorrod H Kearns B Stevens J, et al A review of survival analysis methods used in NICE technology appraisals of cancer treatments: consistency, limitations and areas for improvement. Med Decis Making. 2019; 39(8):899–909. 31707911 10.1177/0272989X19881967

[3] Akaike H. Information theory and an extension of the maximum likelihood principle. In: Parzen E, Tanabe K, Kitagawa G, eds. Selected Papers of Hirotugu Akaike. New York, NY: Springer; 1998 p 199–213.

[4] Schwarz G. Estimating the dimension of a model. Ann Stat. 1978; 6(2):461–4.

[5] Volinsky CT Raftery AE. Bayesian information criterion for censored survival models. Biometrics. 2000;56(1):256–62. 10.1111/j.0006-341x.2000.00256.x 10783804

[6] Latimer N. NICE DSU Technical Support Document 14: Survival Analysis for Economic Evaluations alongside Clinical Trials—Extrapolation with Patient-Level Data. Report by the Decision Support Unit. 2011 Available from: http://www.nicedsu.org.uk.

[7] Bellmunt J De Wit R Vaughn DJ, et al Pembrolizumab as second-line therapy for advanced urothelial carcinoma. N Engl J Med. 2017;376(11):1015–26. 10.1056/NEJMoa1613683 PMC5635424 28212060

[8] Mok TS Cheng Y Zhou X, et al Improvement in overall survival in a randomized study that compared dacomitinib with gefitinib in patients with advanced non–small-cell lung cancer and EGFR-activating mutations. J Clin Oncol. 2018;36(22):2244–50. 10.1200/JCO.2018.78.7994 29864379

[9] Seymour JF Kipps TJ Eichhorst B, et al Venetoclax–rituximab in relapsed or refractory chronic lymphocytic leukemia. N Engl J Med. 2018;378(12):1107–20. 10.1056/NEJMoa1713976 29562156

[10] Von Minckwitz G Procter M De Azambuja E, et al Adjuvant pertuzumab and trastuzumab in early HER2-positive breast cancer. N Engl J Med. 2017;377(2):122–31. 10.1056/NEJMoa1703643 PMC5538020 28581356

[11] Guyot P Ades AE Ouwens MJNM Welton NJ. Enhanced secondary analysis of survival data: reconstructing the data from published Kaplan-Meier survival curves. BMC Med Res Methodol. 2012;12(1):9. 22297116 10.1186/1471-2288-12-9 PMC3313891

[12] Jackson CH. flexsurv: a platform for parametric survival modeling in R. J Stat Softw. 2016;70:i08. 29593450 10.18637/jss.v070.i08 PMC5868723

[13] Cox C. The generalized F distribution: an umbrella for parametric survival analysis. Stat Med. 2008;27(21):4301–12. 10.1002/sim.3292 18407568

[14] Morris TP White IR Crowther MJ. Using simulation studies to evaluate statistical methods. Stat Med. 2019;38(11):2074–102. 10.1002/sim.8086 PMC6492164 30652356

[15] Heinze G Wallisch C Dunkler D. Variable selection: a review and recommendations for the practicing statistician. Biom J. 2018;60(3):431–49. 10.1002/bimj.201700067 PMC5969114 29292533

[16] Vickers A. An evaluation of survival curve extrapolation techniques using long-term observational cancer data [published online 10 20, 2019]. Med Decis Making. 10.1177/0272989X19875950 PMC6900572 31631772

[17] Martina R Abrams K Bujkiewicz S, et al The use of registry data to extrapolate overall survival results from randomised controlled trials. arXiv preprint. 2019; arXiv:1911.05691 [stat.AP].

