# Supplementary material for: Extrapolating Parametric Survival Models in Health Technology Assessment: A Simulation Study
Source: Med Decis Making. 2020 Dec 7;41(1):37–50. doi: 10.1177/0272989X20973201 (PMC7780268; doi:10.1177/0272989X20973201)
Supplement: sj-docx-1-mdm-10.1177_0272989X20973201 – Supplemental material for Extrapolating Parametric Survival Models in Health Technology Assessment: A Simulation Study [file sj-docx-1-mdm-10.1177_0272989X20973201.docx]

Supplementary information

Table S1: Unsuccessful Model Convergence from 10,000 runs

| Drug/ Source Curve | Full Follow Up | Trial Follow-up |
| --- | --- | --- |
| Dacomitinib Exponential | Gen F: <1% | Gen Gamma: 5%  Gen F: 23% |
| Dacomitinib Weibull | (none were unsuccessful) | Gen Gamma: 1%  Gen F: 26% |
| Dacomitinib Gen Gamma | Gen F: <1% | Gen Gamma: 2%  Gen F: 37% |
| Pembrolizumab Exponential | Gen F: <1% | Gen F: 1% |
| Pembrolizumab Weibull | Gen F: <1% | Gen F: 2% |
| Pembrolizumab Gen Gamma | Exponential: 10%  Gompertz: <1%  Gen F: <1% | Gen F: <1% |
| Pertuzumab Exponential | Gen F: <1% | Gen Gamma: 7%  Gen F: 82% |
| Pertuzumab Weibull | Gen F: <1% | Gen Gamma: 8%  Gen F: 92% |
| Pertuzumab Gen Gamma | Gen F: <1% | Gen Gamma: 8%  Gen F: 83% |
| Venetoclax Exponential | Gen F: <1% | Weibull: <1%  Gamma: 2%  Gen Gamma: 12%  Gen F: 51% |
| Venetoclax Weibull | Gompertz: 54%  Gen F: <1% | Gamma: <1%  Gen Gamma: 27%  Gen F: 50% |
| Venetoclax Gen Gamma | Gen Gamma: <1%  Gen F: 4% | Gamma: <1%  Gen Gamma: 27%  Gen F: 50% |

Gen: Generalised. All models not included in this table successfully converged in all runs.

Table S2: Results for trial follow-up scenarios

| Drug/ Trial/ Outcome/ Source distribution | True RMST (+/- 10%) | Measure | Results | | | | | | | | | | | |
| --- | --- | --- | --- | --- | --- | --- | --- | --- | --- | --- | --- | --- | --- | --- |
|  |  |  | Exponential | Weibull | Log-normal | Log-logistic | Gamma | Generalised gamma | Gompertz | Generalised F | Model with lowest AIC | Model with lowest BIC | Model with highest log-likelihood |  |
| Dacomitinib/ ARCHER 1050/ OS/ Exponential | 50.8  (45.7 - 55.9 | Mean [bias]  (5%, 95%)  Median  MSE  MCSE  EmpSE  % in 10% | **51.03 [+0.18, +0%]**  **(43.79, 59.09)**  **50.84 [-0.00, -0%]**  **21.93**  **0.05**  **4.68**  **72.5%** | 50.95 [+0.11, +0%]  (41.79, 61.61)  50.46 [-0.38, -1%]  37.14  0.06  6.09  59.4% | 69.87 [+19.03, +37%]  (59.10, 81.12)  69.74 [+18.89, +37%]  407.27  0.07  6.72  1.4% | 63.86 [+13.02, +26%]  (54.63, 73.83)  63.62 [+12.78, +25%]  203.70  0.06  5.86  8.4% | 50.93 [+0.08, +0%]  (42.53, 60.55)  50.56 [-0.28, -1%]  30.25  0.05  5.50  64.6% | 51.33 [+0.48, +1%]  (35.28, 69.81)  50.96 [+0.12, +0%]  115.16  0.11  10.72  31.2% | 53.13 [+2.29, +5%]  (37.01, 77.35)  50.47 [-0.37, -1%]  166.69  0.13  12.71  29.1% | 60.75 [+9.91, +19%]  (39.24, 80.74)  61.10 [+10.26, +20%]  254.63  0.14  12.51  16.9% | 53.28 [+2.44, +5%]  (39.59, 71.68)  51.94 [+1.09, +2%]  93.55  0.09  9.36  53.6% | 51.51 [+0.67, +1%]  (43.33, 61.05)  50.99 [+0.15, +0%]  39.17  0.06  6.22  68.7% | 57.26 [+6.42, +13%]  (36.10, 79.68)  57.39 [+6.55, +13%]  227.10  0.14  13.63  21.9% |  |
| Dacomitinib/ ARCHER 1050/ OS/ Weibull | 39.3  (35.4 - 43.2) | Mean [bias]  (5%, 95%)  Median  MSE  MCSE  EmpSE  % in 10% | 52.27 [+12.93, +33%]  (45.39, 59.73)  52.05 [+12.72, +32%]  186.18  0.04  4.35  1.2% | **39.59 [+0.25, +1%]**  **(34.40, 46.02)**  **39.19 [-0.14, -0%]**  **13.09**  **0.04**  **3.61**  **74.1%** | 56.60 [+17.27, +44%]  (47.29, 67.03)  56.21 [+16.88, +43%]  334.62  0.06  6.02  0.6% | 51.71 [+12.37, +31%]  (44.46, 59.82)  51.39 [+12.06, +31%]  175.33  0.05  4.71  2.4% | 42.31 [+2.98, +8%]  (36.66, 48.96)  41.94 [+2.61, +7%]  23.00  0.04  3.76  61.6% | 40.90 [+1.57, +4%]  (31.88, 54.52)  39.61 [+0.28, +1%]  52.26  0.07  7.06  45.3% | 34.66 [-4.67, -12%]  (30.88, 39.71)  34.29 [-5.04, -13%]  29.75  0.03  2.81  32.1% | 47.85 [+8.52, +22%]  (33.35, 67.73)  46.25 [+6.92, +18%]  185.82  0.12  10.65  20.4% | 42.93 [+3.60, +9%]  (32.52, 58.25)  41.01 [+1.68, +4%]  78.53  0.08  8.10  51.4% | 42.90 [+3.57, +9%]  (32.61, 57.48)  41.02 [+1.69, +4%]  73.89  0.08  7.82  51.0% | 45.45 [+6.12, +16%]  (32.10, 65.80)  43.36 [+4.03, +10%]  150.44  0.11  10.63  44.9% |  |
| Dacomitinib/ ARCHER 1050/ OS/ Generalised gamma | 35.7  (32.1 - 39.3) | Mean [bias]  (5%, 95%)  Median  MSE  MCSE  EmpSE  % in 10% | 52.54 [+16.81, +47%]  (45.69, 60.04)  52.36 [+16.64, +47%]  301.64  0.04  4.36  0.0% | 40.00 [+4.28, +12%]  (34.70, 46.72)  39.54 [+3.81, +11%]  32.08  0.04  3.71  46.7% | 58.53 [+22.80, +64%]  (48.87, 69.35)  58.24 [+22.51, +63%]  558.69  0.06  6.24  0.0% | 52.72 [+17.00, +48%]  (45.44, 61.19)  52.42 [+16.69, +47%]  312.07  0.05  4.82  0.0% | 43.03 [+7.30, +20%]  (37.38, 49.84)  42.71 [+6.98, +20%]  68.07  0.04  3.85  16.4% | **37.62 [+1.90, +5%]**  **(30.77, 49.81)**  **36.14 [+0.41, +1%]**  **39.59**  **0.06**  **6.00**  **55.1%** | 34.32 [-1.41, -4%]  (30.77, 39.04)  33.97 [-1.75, -5%]  8.84  0.03  2.62  76.3% | 44.54 [+8.81, +25%]  (31.92, 64.40)  42.58 [+6.86, +19%]  182.91  0.13  10.26  20.4% | 39.16 [+3.43, +10%]  (31.32, 54.22)  37.62 [+1.90, +5%]  60.94  0.07  7.01  51.4% | 39.41 [+3.68, +10%]  (31.38, 55.11)  37.67 [+1.94, +5%]  64.73  0.07  7.15  51.0% | 41.08 [+5.35, +15%]  (31.05, 61.25)  37.64 [+1.92, +5%]  122.43  0.10  9.68  44.9% |  |
| Venetoclax/ MURANO/ OS/ Exponential | 169.4 (152.5 - 186.3 | Mean [bias]  (5%, 95%)  Median  MSE  MCSE  EmpSE  % in 10% | **170.04 [+0.63, +0%]**  **(146.76, 193.83)**  **169.59 [+0.18, +0%]**  **202.54**  **0.14**  **14.22**  **77.5%** | 162.14 [-7.27, -4%]  (107.63, 202.72)  166.07 [-3.34, -2%]  879.61  0.29  28.76  46.6% | 186.25 [+16.85, +10%]  (155.47, 210.73)  188.20 [+18.80, +11%]  575.23  0.17  17.07  41.3% | 171.16 [+1.75, +1%]  (129.48, 204.33)  173.62 [+4.21, +2%]  523.56  0.23  22.82  53.8% | 164.49 [-4.91, -3%]  (120.69, 201.50)  166.45 [-2.95, -2%]  625.28  0.25  24.52  49.9% | 174.89 [+5.48, +3%]  (97.86, 216.69)  184.42 [+15.01, +9%]  1344.54  0.39  36.26  26.3% | 151.47 [-17.94, -11%]  (60.68, 221.76)  167.63 [-1.77, -1%]  3736.89  0.58  58.44  13.3% | 183.24 [+13.83, +8%]  (114.41, 215.35)  192.73 [+23.32, +14%]  1265.81  0.47  32.78  12.7% | 168.80 [-0.61, -0%]  (65.28, 216.58)  171.74 [+2.33, +1%]  1229.46  0.35  35.06  58.2% | 168.37 [-1.03, -1%]  (142.23, 197.50)  169.49 [+0.08, +0%]  501.22  0.22  22.37  73.6% | 169.50 [+0.09, +0%]  (64.46, 218.82)  188.49 [+19.08, +11%]  2452.84  0.50  49.53  22.0% |  |
| Venetoclax/ MURANO/ OS/ Weibull | 193.2  (173.9 - 212.5) | Mean [bias]  (5%, 95%)  Median  MSE  MCSE  EmpSE  % in 10% | 170.46 [-22.76, -12%]  (146.87, 193.85)  170.86 [-22.36, -12%]  722.87  0.14  14.32  40.3% | **188.41 [-4.81, -2%]**  **(153.92, 213.39)**  **191.17 [-2.05, -1%]**  **378.06**  **0.19**  **18.84**  **75.4%** | 200.27 [+7.05, +4%]  (179.33, 217.23)  201.48 [+8.26, +4%]  188.79  0.12  11.79  83.9% | 192.02 [-1.20, -1%]  (163.91, 213.96)  193.86 [+0.64, +0%]  249.17  0.16  15.74  81.5% | 187.63 [-5.59, -3%]  (155.23, 212.74)  189.72 [-3.50, -2%]  347.89  0.18  17.80  74.2% | 191.52 [-1.70, -1%]  (149.76, 220.05)  195.68 [+2.46, +1%]  583.31  0.28  24.09  49.0% | 199.25 [+6.03, +3%]  (99.66, 226.28)  213.16 [+19.94, +10%]  1391.68  0.37  36.82  33.6% | 198.79 [+5.57, +3%]  (161.72, 219.04)  204.26 [+11.04, +6%]  534.14  0.31  22.43  34.6% | 190.99 [-2.23, -1%]  (153.53, 223.30)  194.08 [+0.86, +0%]  708.23  0.27  26.52  53.8% | 180.13 [-13.09, -7%]  (149.46, 220.16)  177.18 [-16.04, -8%]  749.56  0.24  24.05  46.5% | 195.15 [+1.93, +1%]  (122.24, 222.91)  203.73 [+10.50, +5%]  1011.93  0.32  31.75  59.7% |  |
| Venetoclax/ MURANO/ OS/ Generalised gamma | 190.3  (171.3-209.3) | Mean [bias]  (5%, 95%)  Median  MSE  MCSE  EmpSE  % in 10% | 171.04 [-19.22, -10%]  (147.51, 194.63)  171.21 [-19.05, -10%]  579.04  0.14  14.47  49.6% | 188.41 [-1.85, -1%]  (153.05, 213.66)  191.29 [+1.02, +1%]  377.25  0.19  19.34  73.4% | 200.45 [+10.18, +5%]  (179.33, 217.55)  201.59 [+11.33, +6%]  248.00  0.12  12.01  74.6% | 192.08 [+1.82, +1%]  (163.20, 214.32)  193.99 [+3.73, +2%]  262.84  0.16  16.11  77.7% | 187.66 [-2.60, -1%]  (154.57, 213.08)  189.86 [-0.41, -0%]  337.91  0.18  18.20  73.2% | **191.67 [+1.41, +1%]**  **(148.58, 220.17)**  **195.91 [+5.64, +3%]**  **567.09**  **0.28**  **23.77**  **45.7%** | 198.25 [+7.99, +4%]  (95.73, 226.32)  213.02 [+22.76, +12%]  1510.62  0.38  38.04  26.0% | 199.22 [+8.96, +5%]  (162.32, 219.19)  204.41 [+14.15, +7%]  523.05  0.30  21.05  28.8% | 190.62 [+0.36, +0%]  (153.05, 223.55)  194.11 [+3.85, +2%]  687.04  0.26  26.21  53.1% | 180.38 [-9.88, -5%]  (150.01, 219.94)  177.26 [-13.00, -7%]  628.47  0.23  23.04  51.8% | 195.26 [+5.00, +3%]  (120.62, 223.41)  203.65 [+13.39, +7%]  1020.21  0.32  31.55  52.0% |  |

AIC: Akaike information criterion; BIC: Bayes information criterion; EmpSE: empirical standard error; IDFS: invasive disease free survival; MCSE: Monte-Carlo standard error; MSE: mean squared error; OS: overall survival; PFS: progression free survival.
Shaded cells indicate model or model selection method with lowest MSE. Bold cells indicate model distribution matches source distribution.

Table S3: Results for complete follow-up scenarios

| Drug/ Trial/ Outcome/ Source distribution | True RMST (+/- 10%) | Measure | Results | | | | | | | | | | | |
| --- | --- | --- | --- | --- | --- | --- | --- | --- | --- | --- | --- | --- | --- | --- |
|  |  |  | Exponential | Weibull | Log-normal | Log-logistic | Gamma | Generalised gamma | Gompertz | Generalised F | Model with lowest AIC | Model with lowest BIC | Model with highest log-likelihood |  |
| Dacomitinib/ ARCHER 1050/ OS/ Exponential | 50.8  (45.7 - 55.9 | Mean [bias]  (5%, 95%)  Median  MSE  MCSE  EmpSE  % in 10% | **50.75 [-0.09, -0%]**  **(45.79, 55.77)**  **50.73 [-0.12, -0%]**  **9.05**  **0.03**  **3.01**  **90.7%** | 50.76 [-0.09, -0%]  (45.74, 55.79)  50.73 [-0.12, -0%]  9.30  0.03  3.05  90.3% | 51.12 [+0.28, +1%]  (46.73, 55.57)  51.11 [+0.26, +1%]  7.25  0.03  2.68  94.3% | 53.08 [+2.24, +4%]  (48.35, 57.86)  53.06 [+2.22, +4%]  13.23  0.03  2.87  83.5% | 50.76 [-0.08, -0%]  (45.76, 55.78)  50.73 [-0.12, -0%]  9.20  0.03  3.03  90.6% | 50.75 [-0.09, -0%]  (45.71, 55.87)  50.71 [-0.13, -0%]  9.53  0.03  3.09  90.1% | 50.79 [-0.05, -0%]  (45.71, 55.92)  50.76 [-0.08, -0%]  9.62  0.03  3.10  89.9% | 50.65 [-0.19, -0%]  (45.57, 55.80)  50.61 [-0.23, -0%]  9.73  0.03  3.11  89.7% | 50.76 [-0.09, -0%]  (45.73, 55.84)  50.73 [-0.12, -0%]  9.44  0.03  3.07  90.3% | 50.75 [-0.09, -0%]  (45.78, 55.79)  50.73 [-0.11, -0%]  9.13  0.03  3.02  90.6% | 50.67 [-0.17, -0%]  (45.60, 55.83)  50.64 [-0.20, -0%]  9.76  0.03  3.12  89.9% |  |
| Dacomitinib/ ARCHER 1050/ OS/ Weibull | 39.3  (35.4 - 43.2) | Mean [bias]  (5%, 95%)  Median  MSE  MCSE  EmpSE  % in 10% | 38.89 [-0.44, -1%]  (36.10, 41.69)  38.88 [-0.45, -1%]  3.13  0.02  1.71  97.4% | **39.30 [-0.03, -0%]**  **(36.35, 42.25)**  **39.28 [-0.05, -0%]**  **3.29**  **0.02**  **1.81**  **97.0%** | 41.38 [+2.05, +5%]  (38.29, 44.51)  41.35 [+2.02, +5%]  7.76  0.02  1.89  84.2% | 43.21 [+3.88, +10%]  (40.02, 46.45)  43.19 [+3.86, +10%]  18.87  0.02  1.96  51.8% | 39.27 [-0.07, -0%]  (36.33, 42.22)  39.25 [-0.09, -0%]  3.27  0.02  1.81  97.1% | 39.30 [-0.03, -0%]  (36.35, 42.29)  39.28 [-0.05, -0%]  3.30  0.02  1.82  97.0% | 39.13 [-0.20, -1%]  (36.21, 42.10)  39.11 [-0.22, -1%]  3.30  0.02  1.81  97.0% | 39.31 [-0.02, -0%]  (36.36, 42.30)  39.28 [-0.05, -0%]  3.36  0.02  1.83  96.8% | 39.29 [-0.04, -0%]  (36.34, 42.26)  39.27 [-0.06, -0%]  3.34  0.02  1.83  96.9% | 39.28 [-0.05, -0%]  (36.33, 42.24)  39.26 [-0.07, -0%]  3.29  0.02  1.81  97.0% | 39.30 [-0.03, -0%]  (36.35, 42.30)  39.28 [-0.05, -0%]  3.36  0.02  1.83  96.9% |  |
| Dacomitinib/ ARCHER 1050/ OS/ Generalised gamma | 35.7  (32.1 - 39.3) | Mean [bias]  (5%, 95%)  Median  MSE  MCSE  EmpSE  % in 10% | 35.48 [-0.24, -1%]  (33.26, 37.68)  35.50 [-0.23, -1%]  1.88  0.01  1.35  99.0% | 35.55 [-0.17, -0%]  (33.27, 37.84)  35.55 [-0.17, -0%]  1.98  0.01  1.40  98.8% | 38.65 [+2.92, +8%]  (36.02, 41.33)  38.63 [+2.90, +8%]  11.13  0.02  1.61  66.1% | 40.77 [+5.04, +14%]  (38.11, 43.37)  40.77 [+5.04, +14%]  27.93  0.02  1.59  17.6% | 35.71 [-0.02, -0%]  (33.41, 38.00)  35.72 [-0.01, -0%]  1.97  0.01  1.40  98.9% | **35.74 [+0.01, +0%]**  **(33.44, 38.02)**  **35.74 [+0.02, +0%]**  **1.97**  **0.01**  **1.40**  **98.8%** | 35.61 [-0.12, -0%]  (33.31, 37.89)  35.61 [-0.12, -0%]  1.97  0.01  1.40  98.8% | 35.80 [+0.08, +0%]  (33.46, 38.11)  35.78 [+0.05, +0%]  2.45  0.02  1.56  97.6% | 35.74 [+0.01, +0%]  (33.41, 38.06)  35.73 [+0.00, +0%]  2.20  0.01  1.48  98.6% | 35.64 [-0.08, -0%]  (33.34, 37.92)  35.65 [-0.08, -0%]  1.97  0.01  1.40  98.8% | 35.81 [+0.08, +0%]  (33.47, 38.12)  35.78 [+0.05, +0%]  2.45  0.02  1.56  98.5% |  |
| Venetoclax/ MURANO/ OS/ Exponential | 169.4 (152.5 - 186.3 | Mean [bias]  (5%, 95%)  Median  MSE  MCSE  EmpSE  % in 10% | **169.19 [-0.22, -0%]**  **(162.24, 175.64)**  **169.37 [-0.04, -0%]**  **16.43**  **0.04**  **4.05**  **100.0%** | 169.49 [+0.08, +0%]  (160.02, 178.74)  169.57 [+0.16, +0%]  32.42  0.06  5.69  99.6% | 158.84 [-10.57, -6%]  (148.10, 169.78)  158.81 [-10.60, -6%]  154.93  0.07  6.57  83.2% | 168.04 [-1.37, -1%]  (157.32, 178.59)  168.05 [-1.36, -1%]  43.73  0.06  6.47  98.8% | 169.48 [+0.07, +0%]  (160.26, 178.56)  169.51 [+0.10, +0%]  30.61  0.06  5.53  99.8% | 169.54 [+0.13, +0%]  (159.99, 178.77)  169.66 [+0.25, +0%]  32.76  0.06  5.72  99.6% | 169.77 [+0.37, +0%]  (161.23, 177.84)  169.88 [+0.47, +0%]  25.60  0.05  5.05  99.9% | 170.07 [+0.66, +0%]  (160.36, 179.42)  170.13 [+0.73, +0%]  38.18  0.06  6.14  99.3% | 169.59 [+0.18, +0%]  (160.82, 178.33)  169.65 [+0.24, +0%]  30.56  0.06  5.53  99.6% | 169.32 [-0.08, -0%]  (161.88, 176.39)  169.43 [+0.02, +0%]  20.01  0.04  4.47  99.8% | 170.08 [+0.67, +0%]  (160.33, 179.38)  170.18 [+0.78, +0%]  37.77  0.06  6.11  99.5% |  |
| Venetoclax/ MURANO/ OS/ Weibull | 193.2  (173.9 - 212.5) | Mean [bias]  (5%, 95%)  Median  MSE  MCSE  EmpSE  % in 10% | 217.41 [+24.19, +13%]  (213.53, 220.83)  217.51 [+24.29, +13%]  589.91  0.02  2.23  2.2% | **193.36 [+0.14, +0%]**  **(184.81, 201.45)**  **193.46 [+0.24, +0%]**  **25.87**  **0.05**  **5.08**  **100.0%** | 186.03 [-7.19, -4%]  (174.08, 197.69)  186.02 [-7.20, -4%]  102.30  0.07  7.11  95.3% | 196.24 [+3.02, +2%]  (186.04, 205.81)  196.36 [+3.14, +2%]  45.79  0.06  6.05  99.8% | 193.52 [+0.30, +0%]  (185.62, 201.15)  193.51 [+0.29, +0%]  22.67  0.05  4.75  100.0% | 193.38 [+0.16, +0%]  (184.79, 201.61)  193.41 [+0.19, +0%]  26.34  0.05  5.13  100.0% | 209.45 [+16.23, +8%]  (204.32, 214.01)  209.60 [+16.38, +8%]  272.25  0.04  2.98  39.0% | 193.86 [+0.64, +0%]  (185.21, 202.00)  193.81 [+0.59, +0%]  31.37  0.06  5.56  99.6% | 193.43 [+0.21, +0%]  (184.84, 201.48)  193.43 [+0.21, +0%]  29.60  0.05  5.44  99.8% | 193.21 [-0.01, -0%]  (184.78, 201.19)  193.26 [+0.04, +0%]  25.57  0.05  5.06  100.0% | 193.85 [+0.63, +0%]  (185.20, 202.00)  193.81 [+0.59, +0%]  31.38  0.06  5.57  99.7% |  |
| Venetoclax/ MURANO/ OS/ Generalised gamma | 190.3  (171.3-209.3) | Mean [bias]  (5%, 95%)  Median  MSE  MCSE  EmpSE  % in 10% | 190.21 [-0.05, -0%]  (185.77, 194.23)  190.33 [+0.07, +0%]  6.60  0.03  2.57  100.0% | 192.22 [+1.95, +1%]  (181.72, 202.27)  192.33 [+2.07, +1%]  43.19  0.06  6.28  99.7% | 175.92 [-14.35, -8%]  (163.05, 189.09)  175.86 [-14.41, -8%]  269.75  0.08  7.99  71.3% | 191.83 [+1.56, +1%]  (180.14, 203.10)  191.96 [+1.70, +1%]  51.64  0.07  7.01  99.4% | 188.37 [-1.90, -1%]  (178.25, 198.48)  188.44 [-1.82, -1%]  41.81  0.06  6.18  99.7% | **190.44 [+0.17, +0%]**  **(181.72, 199.05)**  **190.49 [+0.22, +0%]**  **29.02**  **0.05**  **5.38**  **99.9%** | 199.36 [+9.09, +5%]  (192.18, 206.08)  199.47 [+9.21, +5%]  100.52  0.04  4.23  99.4% | 191.98 [+1.72, +1%]  (182.13, 201.60)  190.98 [+0.72, +0%]  84.21  0.09  9.01  93.5% | 191.30 [+1.03, +1%]  (181.81, 200.97)  190.70 [+0.44, +0%]  61.17  0.08  7.75  98.5% | 191.30 [+1.04, +1%]  (181.80, 202.88)  190.59 [+0.33, +0%]  45.92  0.07  6.70  99.6% | 191.68 [+1.41, +1%]  (181.89, 201.12)  190.82 [+0.55, +0%]  77.68  0.09  8.70  97.7% |  |

AIC: Akaike information criterion; BIC: Bayes information criterion; EmpSE: empirical standard error; IDFS: invasive disease free survival; MCSE: Monte-Carlo standard error; MSE: mean squared error; OS: overall survival; PFS: progression free survival.
Shaded cells indicate model or model selection method with lowest MSE. Bold cells indicate model distribution matches source distribution.

Table S4: Results for complete follow-up scenarios

| Drug/ Trial/ Outcome/ Source distribution | True RMST (+/- 10%) | Measure | Results | | | | | | | | | | | |
| --- | --- | --- | --- | --- | --- | --- | --- | --- | --- | --- | --- | --- | --- | --- |
|  |  |  | Exponential | Weibull | Log-normal | Log-logistic | Gamma | Generalised gamma | Gompertz | Generalised F | Model with lowest AIC | Model with lowest BIC | Model with highest log-likelihood |  |
| Pembrolizumab/ KEYNOTE 045/ PFS/ Exponential | 5.6 (5.0 - 6.2) | Mean [bias]  (5%, 95%)  Median  MSE  MCSE  EmpSE  % within 10% | **5.60 [+0.00, +0%]**  **(5.06, 6.18)**  **5.59 [-0.01, -0%]**  **0.12**  **0.00**  **0.34**  **89.9%** | 5.60 [+0.00, +0%]  (5.06, 6.19)  5.59 [-0.01, -0%]  0.12  0.00  0.34  89.9% | 7.14 [+1.54, +28%]  (6.19, 8.28)  7.08 [+1.48, +26%]  2.81  0.01  0.65  4.4% | 8.44 [+2.84, +51%]  (7.32, 9.72)  8.39 [+2.79, +50%]  8.60  0.01  0.73  0.0% | 5.60 [+0.00, +0%]  (5.06, 6.18)  5.59 [-0.01, -0%]  0.12  0.00  0.34  89.9% | 5.60 [+0.00, +0%]  (5.06, 6.18)  5.59 [-0.01, -0%]  0.12  0.00  0.34  89.9% | 5.60 [+0.00, +0%]  (5.06, 6.19)  5.59 [-0.01, -0%]  0.12  0.00  0.34  89.9% | 5.61 [+0.01, +0%]  (5.06, 6.19)  5.60 [-0.00, -0%]  0.29  0.01  0.54  89.6% | 5.61 [+0.01, +0%]  (5.06, 6.19)  5.59 [-0.01, -0%]  0.29  0.01  0.54  89.9% | 5.61 [+0.01, +0%]  (5.06, 6.19)  5.59 [-0.01, -0%]  0.29  0.01  0.54  89.8% | 5.61 [+0.01, +0%]  (5.06, 6.19)  5.60 [-0.00, -0%]0.29  0.01  0.54  89.7% |  |
| Pembrolizumab/ KEYNOTE 045/ PFS/ Weibull | 5.8 (5.2 - 6.4) | Mean [bias]  (5%, 95%)  Median  MSE  MCSE  EmpSE  % within 10% | 5.84 [-0.00, -0%]  (5.18, 6.52)  5.83 [-0.01, -0%]  0.17  0.00  0.41  84.9% | **5.84 [-0.00, -0%]**  **(5.18, 6.52)**  **5.83 [-0.01, -0%]**  **0.17**  **0.00**  **0.41**  **84.8%** | 8.26 [+2.42, +41%]  (6.89, 9.89)  8.19 [+2.34, +40%]  6.68  0.01  0.92  0.9% | 9.93 [+4.09, +70%]  (8.40, 11.60)  9.88 [+4.04, +69%]  17.64  0.01  0.98  0.0% | 5.84 [-0.00, -0%]  (5.18, 6.52)  5.83 [-0.01, -0%]  0.17  0.00  0.41  84.9% | 5.84 [-0.00, -0%]  (5.18, 6.52)  5.83 [-0.01, -0%]  0.17  0.00  0.41  84.9% | 5.96 [+0.12, +2%]  (5.24, 6.74)  5.94 [+0.10, +2%]  0.22  0.00  0.45  80.0% | 5.85 [+0.01, +0%]  (5.19, 6.53)  5.84 [-0.01, -0%]  0.17  0.00  0.41  84.7% | 5.85 [+0.01, +0%]  (5.18, 6.53)  5.84 [-0.00, -0%]  0.17  0.00  0.41  84.6% | 5.85 [+0.01, +0%]  (5.18, 6.54)  5.84 [-0.00, -0%]  0.17  0.00  0.41  84.5% | 5.85 [+0.01, +0%]  (5.19, 6.53)  5.84 [-0.01, -0%]  0.17  0.00  0.41  84.9% |  |
| Pembrolizumab/ KEYNOTE 045/ PFS/ Generalised gamma | 12.0  (10.8 - 13.2) | Mean [bias]  (5%, 95%)  Median  MSE  MCSE  EmpSE  % within 10% | 18.83 [+6.83, +57%]  (8.38, 41.27)  13.48 [+1.48, +12%]  559.15  0.24  22.64  20.4% | 11.35 [-0.65, -5%]  (7.58, 17.53)  10.64 [-1.36, -11%]  11.33  0.03  3.30  26.8% | 8.64 [-3.36, -28%]  (6.57, 11.19)  8.51 [-3.49, -29%]  13.34  0.01  1.43  7.2% | 8.42 [-3.58, -30%]  (6.59, 10.56)  8.33 [-3.66, -31%]  14.28  0.01  1.22  3.4% | 20.05 [+8.05, +67%]  (8.47, 52.28)  14.19 [+2.19, +18%]  411.20  0.19  18.61  20.4% | **12.08 [+0.08, +1%]**  **(8.71, 15.95)**  **11.95 [-0.05, -0%]**  **4.89**  **0.02**  **2.21**  **41.0%** | 8.43 [-3.57, -30%]  (6.58, 10.88)  8.22 [-3.77, -31%]  14.90  0.01  1.47  4.0% | 12.27 [+0.27, +2%]  (8.84, 16.23)  12.13 [+0.13, +1%]  5.18  0.02  2.26  40.6% | 12.14 [+0.14, +1%]  (8.75, 16.09)  12.00 [+0.00, +0%]  5.03  0.02  2.24  40.8% | 12.09 [+0.09, +1%]  (8.71, 15.98)  11.96 [-0.04, -0%]  4.93  0.02  2.22  41.0% | 12.26 [+0.26, +2%]  (8.84, 16.22)  12.12 [+0.13, +1%]  5.17  0.02  2.26  40.6% |  |
| Pertuzumab/ APHINITY/ IDFS/ Exponential | 376.8  (339.1 - 414.5) | Mean [bias]  (5%, 95%)  Median  MSE  MCSE  EmpSE  % within 10% | **376.81 [-0.04, -0%]**  **(370.99, 382.54)**  **376.83 [-0.01, -0%]**  **12.33**  **0.04**  **3.51**  **100.0%** | 376.86 [+0.02, +0%]  (369.63, 383.98)  376.90 [+0.05, +0%]  19.00  0.04  4.36  100.0% | 348.23 [-28.62, -8%]  (341.03, 355.38)  348.25 [-28.60, -8%]  837.86  0.04  4.35  98.1% | 368.16 [-8.68, -2%]  (360.43, 375.76)  368.19 [-8.65, -2%]  96.91  0.05  4.64  100.0% | 376.85 [+0.00, +0%]  (369.90, 383.63)  376.90 [+0.06, +0%]  17.41  0.04  4.17  100.0% | 376.89 [+0.04, +0%]  (369.42, 384.26)  376.88 [+0.04, +0%]  20.14  0.04  4.49  100.0% | 376.95 [+0.11, +0%]  (369.94, 383.87)  376.93 [+0.09, +0%]  18.27  0.04  4.27  100.0% | 377.23 [+0.39, +0%]  (369.69, 384.67)  377.25 [+0.41, +0%]  20.71  0.05  4.53  99.5% | 376.93 [+0.08, +0%]  (369.92, 383.83)  376.95 [+0.11, +0%]  17.97  0.04  4.24  100.0% | 376.81 [-0.03, -0%]  (370.89, 382.63)  376.84 [+0.00, +0%]  12.93  0.04  3.60  100.0% | 377.22 [+0.37, +0%]  (369.65, 384.64)  377.24 [+0.40, +0%]  20.80  0.05  4.55  100.0% |  |
| Pertuzumab/ APHINITY/ IDFS/ Weibull | 338.7 (304.8 - 372.6) | Mean [bias]  (5%, 95%)  Median  MSE  MCSE  EmpSE  % within 10% | 325.71 [-12.95, -4%]  (320.13, 331.25)  325.72 [-12.94, -4%]  179.16  0.03  3.39  100.0% | **338.62 [-0.03, -0%]**  **(331.70, 345.54)**  **338.63 [-0.02, -0%]**  **18.02**  **0.04**  **4.24**  **100.0%** | 315.05 [-23.60, -7%]  (308.58, 321.54)  315.06 [-23.60, -7%]  572.83  0.04  3.97  99.4% | 331.08 [-7.57, -2%]  (324.02, 338.01)  331.12 [-7.54, -2%]  75.68  0.04  4.29  100.0% | 336.44 [-2.22, -1%]  (329.74, 343.12)  336.44 [-2.22, -1%]  21.77  0.04  4.10  100.0% | 338.66 [+0.00, +0%]  (331.46, 345.83)  338.69 [+0.03, +0%]  19.10  0.04  4.37  99.5% | 336.22 [-2.44, -1%]  (329.33, 343.12)  336.22 [-2.43, -1%]  23.66  0.04  4.21  100.0% | 338.87 [+0.21, +0%]  (331.61, 346.07)  338.89 [+0.23, +0%]  19.34  0.04  4.39  99.5% | 338.45 [-0.21, -0%]  (331.30, 345.55)  338.48 [-0.17, -0%]  18.94  0.04  4.35  100.0% | 338.30 [-0.36, -0%]  (331.22, 345.28)  338.33 [-0.32, -0%]  18.50  0.04  4.29  100.0% | 338.82 [+0.17, +0%]  (331.57, 346.00)  338.85 [+0.19, +0%]  19.30  0.04  4.39  100.0% |  |
| Pertuzumab/ APHINITY/ IDFS/ Generalised gamma | 333.5 (300.2 -  366.9) | Mean [bias]  (5%, 95%)  Median  MSE  MCSE  EmpSE  % within 10% | 317.63 [-15.90, -5%]  (312.13, 323.02)  317.66 [-15.88, -5%]  263.72  0.03  3.32  100.0% | 332.79 [-0.74, -0%]  (325.93, 339.64)  332.78 [-0.75, -0%]  17.94  0.04  4.17  100.0% | 310.18 [-23.35, -7%]  (303.85, 316.55)  310.12 [-23.41, -7%]  560.09  0.04  3.85  99.6% | 326.22 [-7.31, -2%]  (319.35, 333.14)  326.22 [-7.31, -2%]  70.85  0.04  4.17  100.0% | 329.91 [-3.62, -1%]  (323.28, 336.49)  329.88 [-3.65, -1%]  29.35  0.04  4.03  100.0% | **333.52 [-0.01, -0%]**  **(326.48, 340.59)**  **333.55 [+0.02, +0%]**  **18.39**  **0.04**  **4.29**  **100.0%** | 331.09 [-2.44, -1%]  (324.25, 337.95)  331.14 [-2.39, -1%]  23.21  0.04  4.16  100.0% | 333.72 [+0.19, +0%]  (326.65, 340.83)  333.74 [+0.21, +0%]  18.74  0.04  4.33  99.7% | 333.22 [-0.31, -0%]  (326.15, 340.41)  333.25 [-0.28, -0%]  18.80  0.04  4.33  100.0% | 332.76 [-0.77, -0%]  (325.86, 339.68)  332.76 [-0.78, -0%]  18.33  0.04  4.21  100.0% | 333.67 [+0.14, +0%]  (326.57, 340.77)  333.69 [+0.16, +0%]  18.69  0.04  4.32  100.0% |  |

AIC: Akaike information criterion; BIC: Bayes information criterion; EmpSE: empirical standard error; IDFS: invasive disease free survival; MCSE: Monte-Carlo standard error; MSE: mean squared error; OS: overall survival; PFS: progression free survival.
Shaded cells indicate model or model selection method with lowest MSE. Bold cells indicate model distribution matches source distribution.

Table S5: Overview of each scenario

| Scenario |  | Pembrolizumab | Dacomitinib | Venetoclax | Pertuzumab |
| --- | --- | --- | --- | --- | --- |
|  | Parameters |  |  |  |  |
| Exponential | Rate  RMST | 0.17858  5.6 | 0.01903  50.8 | 0.00309  169.4 | 0.00178  376.8 |
| Weibull | Shape  Scale  RMST | 0.86756  5.43792  5.8 | 1.44871  43.37962  39.3 | 0.70361  956.33416  193.2 | 1.10627  438.04256  338.7 |
| Generalised gamma | Mu  Sigma  Q  RMST | 0.67506  1.12759  -0.86757  12.0 | 3.82669  0.51876  1.51244  35.7 | 6.65630  0.67242  2.16067  190.3 | 6.06286  0.87454  1.03747  333.5 |

Figure S1: Distribution of curve selection


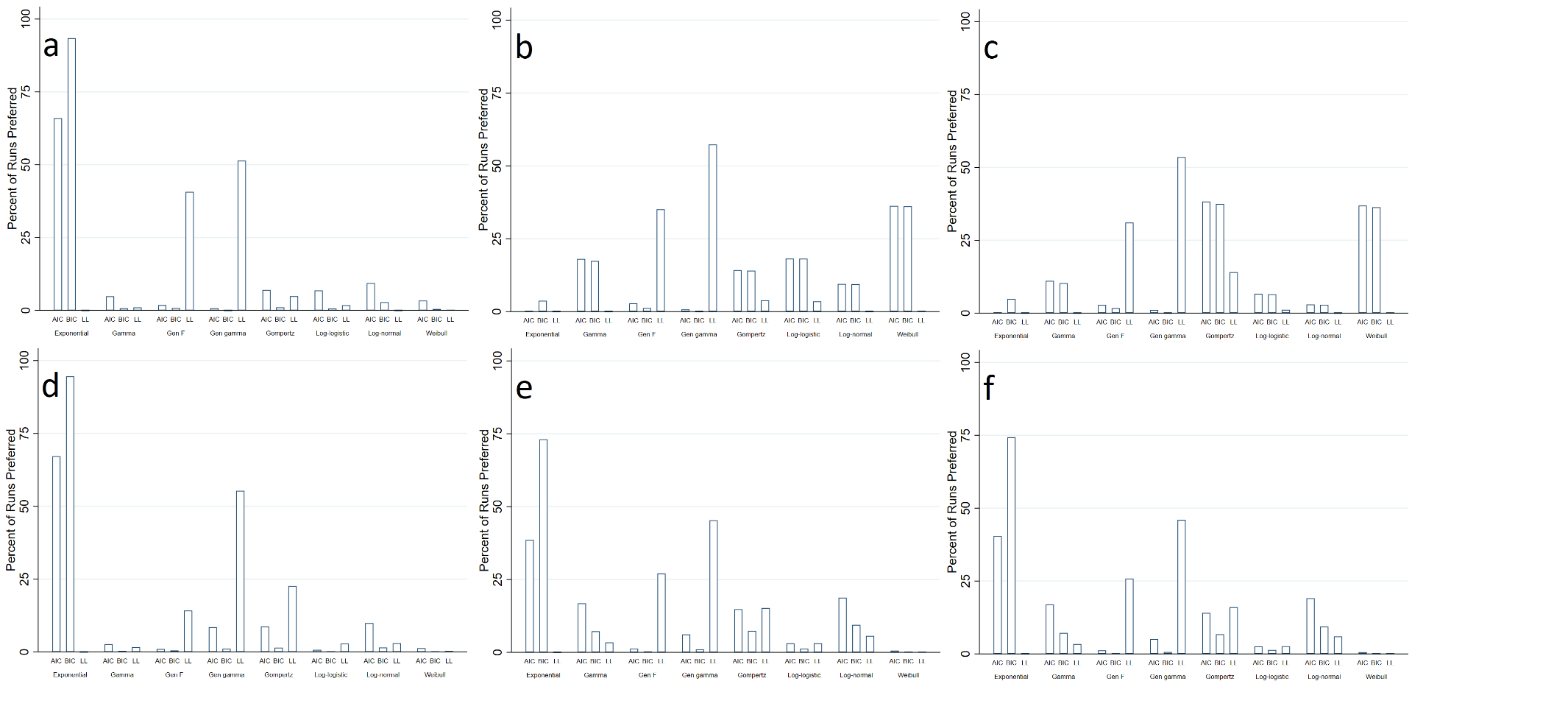


a: exponential dacomitinib scenario; b: Weibull dacomitinib scenario; c: generalised gamma dacomitinib scenario; d: exponential venetoclax scenario; e: Weibull venetoclax scenario; f: generalised gamma venetoclax scenario.
